# Rational Design of a Fluorescent Chromophore as a Calcium Receptor via DFT and Multivariate Approaches

**DOI:** 10.3390/molecules27196248

**Published:** 2022-09-22

**Authors:** Leila Narimani, Vannajan Sanghiran Lee, Yatimah Alias, Ninie Suhana Manan, Pei Meng Woi

**Affiliations:** 1 Chemistry Department, Faculty of Science, Universiti Malaya, Kuala Lumpur 50603, Malaysia; 2 Centre of Ionic Liquids (UMCiL), Chemistry Department, Faculty of Science, Universiti Malaya, Kuala Lumpur 50603, Malaysia

**Keywords:** fluorescein diglycolic acid, calcium, multivariate, DFT, principal component analysis

## Abstract

Computational and experimental approaches were adopted to utilize a chromophore diglycolic functionalized fluorescein derivative as a Ca^2+^ receptor. Fluorescein diglycolic acid (Fl-DGA, **1**) was synthesized and used in multivariate determination of Ca^2+^ and K^+^. Full-structure computation shows that the complexes of **1** and Ca^2+^ have comparable energies regardless of additional interaction with lactone moiety. The initial formation of diglycolic-Ca^2+^ complex followed by macrocyclization is thermodynamically disfavored. A U-shaped pre-organized **1** allows Ca^2+^ to interact simultaneously with diglycolic and lactone motifs. Both motifs actively participate in Ca^2+^ recognition and the eleven methylene units in the undecyl arm provides excellent flexibility for reorganization and optimum interaction. Principal component analysis (PCA) of computational molecular properties reveals a simple method in evaluating motifs for cation recognition. Fragment models support full-structure results that negative charge causes significant structural changes, but do not reproduce the full extent of C-O bond breaking observed in the latter. Experimental optical responses show that **1** is selective towards Ca^2+^ and discriminates against K^+^ and Mg^2+^. PCA of emission intensities affords distinct clusters of 0.01, 0.1 and 1 mM Ca^2+^ and K^+^, and suggests applicability of this technique for simultaneous determination of cationic plant macronutrients in precision agriculture and a wide variety of other applications.

## 1. Introduction

Fluorescein derivatives are one of the most widely used chromophores in the labelling of biomolecules. The hydrophilic nature of fluorescent dyes greatly contributes to its compatibility with biological systems such as in enzyme detection and cell tracking. Fluorescent sensors have successfully been utilized in imaging cell structures and for analyzing biophysical processes: for example, in the detection of Alzheimer’s disease and the activity of certain enzymes, as well as pH and the concentration of certain metal ions [1,2,3,4]. Due to its versatility and widespread utility as a fluorescent probe, numerous synthetic routes have been reported on xanthene dyes and fluorescein derivatives [5,6]. The xanthene core has also received considerable interest from the computational community, helping to highlight the importance of its numerous forms [7].

The development of optical sensors for metal cations has attracted much attention due to their wide applications, which provide reliable and highly sensitive detection of bioanalytes and chemical species [8,9]. Calcium and magnesium are of significant interest in biological analysis due to their role as essential nutrients in human body for bone formation and as intercellular messengers [10]. In precision agriculture, the two alkaline earth metals, in addition to potassium, are macronutrients and thus consumed in large quantities by plants. Potassium is required for fruit formation and carbohydrate metabolism. Calcium participates in cell division, respiration, and nitrogen metabolism, whereas magnesium plays a critical role in chlorophyll production and the activation of plant enzymes [11,12]. To avoid excessive fertilization, which gives rise to a greater extent of fertilizer run-off that can severely contaminate sources of fresh water and cause economic loss, such macronutrients need to be monitored [13,14]. For the divalent cations- Ca^2+^ and Mg^2+^, the conventional sensing method often leads to discrepancies between calibration equations and actual sensor characteristics attributed to erroneous or inaccurate sensor measurement [15,16]. This is the main cause of sensor signal drift error. Another common cause of sensor signal error is false signal due to interfering ions that compete with the target analyte. Thus, algorithms that compensate for the signal error due to these reasons are required.

The principal component analysis (PCA) method has been successfully implemented to determine chemical concentrations, standard formulations, industrial quality control and detection of food adulteration [17,18]. In this approach, variations in the variables are transformed into a few important components with the highest eigenvalues [19]. The score plot of the first two or three components typically afford distinct clusters that represent the populations of chemicals or standard formulations. We aim to simplify the complexity in the discrimination of these divalent cations by transforming the high-dimensional data into fewer dimensions, while retaining trends and patterns which will act as summaries of quantification features.

We earlier demonstrated a rational design of cation receptors using the DFT method [20,21,22]. Chemical selectivity could be described by understanding the nature of interaction between cations and commonly employed functionalities or motifs that include heteroatoms and various forms of carbonyls [23]. Breaking the carbon-heteroatom bond in lactone or lactam core presumably contributes to fluorescence enhancement in fluorescein derivatives. This is supported by the increase in fluorescence activity in alkaline solutions [24,25]. The onset of the bond-breaking event could be estimated from computational activation energy [26]. The dramatic reduction in the activation barrier has been rationalized in terms of the weakening of the C-O bond due to anionic substituent (especially alkoxy), the lack of ion-pair formation due to the chelation of the cation counter ion, formation of a stable intermediate complex and the absence of solvation [27,28,29].

**Scheme 1:**
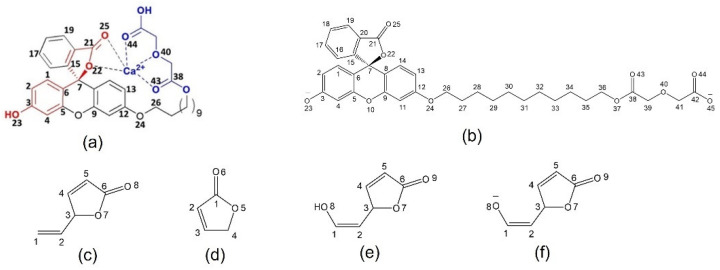

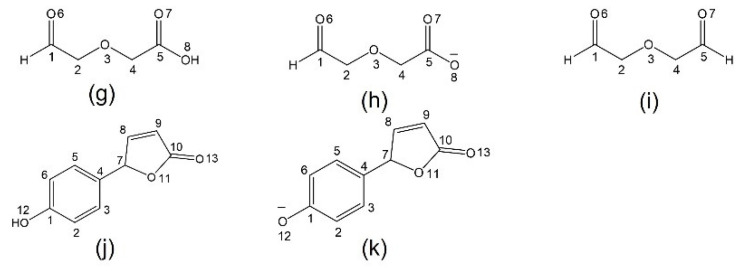
Models with atom numbering systems; (**a**) **1** (red is chromophore core; and blue is Ca^2+^ receptor), (**b**) **1a**, (**c**) **2**, (**d**) **2a**, (**e**) **3**, (**f**) **3a**, (**g**) **4**, (**h**) **4a**, (**i**) **4b**, (**j**) **5** and (**k**) **5a**.

In this study, our main interest is the facile functionalization of fluorescein with a Ca^2+^ receptor, which, when attached to an extended undecyl arm, provides free movement for binding with a cation and interacting with the chromophore core and its fragments (Scheme 1). We describe a rational design of a fluorescent Ca^2+^ receptor by employing density functional theory (DFT) computation to examine chromophore and receptor models. In the proposed fluorophore, the chromophore model comprises phenol with ene-lactone ring at the α-carbon and the receptor model is a diglycolic acid moiety.

Structural characteristics and energetics of the models and its complexes with cations are examined to elucidate cation recognition. In particular, the lactone C-O bond distance and the cation-oxygen bond distances are closely monitored. We are motivated to determine the roles of the fluorescein core and the diglycolic receptor in Ca^2+^ recognition. We explore the potential of utilizing computational molecular properties in the multivariate determination of cations. Electronic properties such as magnetic shielding coefficients, atomic valence, total overlap population (TOP) [30,31], atomic charge and bonding orbital characters are analyzed to extract useful trends that indicate preferential binding to cations and utilized as variables in multivariate analysis. We hope to gain insights into the effect of anion on the structure of fluorescein derivatives and its influence on lactone motif and cation selectivity. Next, a suitable functionalized fluorophore, fluorescein diglycolic acid derivative **1**, subsequently abbreviated as Fl-DGA, is synthesized and its fluorescence emission responses with Ca^2+^, Mg^2+^ and K^+^ are studied. The emission intensities at wavelengths in the neighborhood of the maximum emission are taken as variables in PCA. Earlier, we attempted to use fragments to represent motifs in recognition molecules. Now that we have experimental and full-structure computational data, we can focus on the question of whether fragmented models represent reality. We seek the answer to this question in this report.

## 2. Results and Discussion

### 2.1. Full-Structure Computations

**Figure 1:**
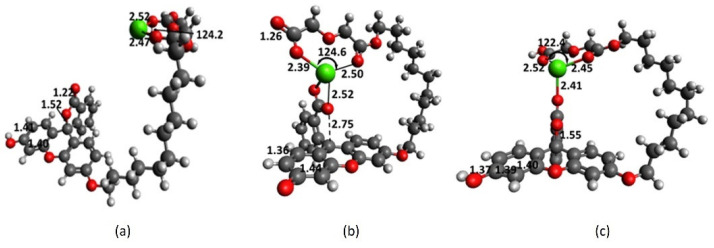
Complexes of **1** and **1a** with Ca^2+^ in methanol; (**a**) Non-interacting **1**-Ca^2+^, (**b**) strongly interacting **1a**-Ca^2+^, and (**c**) interacting **1**-Ca^2+^.

Optimized geometries of full fluorophore-cation complexes provide the most complete three-dimensional pictures to account for contribution from various motifs in cation recognition. The effect of cation on the structure of fluorophore could be visually inspected, and vice versa, the influence of motifs in the fluorophore to cation stabilization can be examined. The optimized geometry of **1** in methanol is obtained by removing Ca^2+^ from the interacting complex (Figure 1c). Ca^2+^ is bound to **1** through the diglycolic arm, which does not interact with the lactone core, as in Figure 1a, by 18.1 kcal mol^−1^. However, Ca^2+^ is bound in Figure 1c by only 17.7 kcal mol^−1^. This means the macrocyclization process that transforms the non-interacting complex in Figure 1a to the interacting one in Figure 1c is disfavored thermodynamically by 0.4 kcal mol^−1^. Considering the uncertainties in the employed level of theory and IEF-PCM model, it is reasonable to assume that the non-interacting and interacting complexes have similar energies.

In the absence of interaction between cation and the chromophore core, the lactone C_7_-O_22_ bond distance in methanol is 1.52 Å (Figure 1a). The C-C bonds in the xanthene ring system has approximately identical bond distances, i.e., 1.39 Å in C_1_-C_2_ and 1.40 Å in C_2_-C_3_.

The spirolactone ring on C_7_ is perpendicular to the planar xanthene framework. The undecyl arm moves above the plane with the following turning points and dihedral angles that position the diglycolic moiety on the right side above the lactone ring; C_27_ (80°), C_32_ (66°) and C_36_ (0°). The diglycolic group is planar and characterized by having three oxygens interact favorably with Ca^2+^ with the following bond distances: 2.47, 2.58 and 2.52 Å. The bond angle involving Ca^2+^ and two carbonyl oxygens is 124.2°. The Ca-O bond distance is indicative of cation binding strength and exhibits the following trend: ester carbonyl (O_43_) > acid carbonyl (O_44_) > ether linkage (O_40_).

Deprotonations of phenol and diglycolic moieties gives rise to dianion **1a** in Figure 1b which is characterized by having both the chromophore and receptor strongly interacting with Ca^2+^. The C-C bonds in the xanthene framework show distinct single and double bond characteristics: the C_1_-C_2_ bond distance shrinks to 1.36 Å while the C_2_-C_3_ bond elongates to 1.44 Å. More importantly, the lactone C_7_-O_22_ bond with a bond distance of 2.75 Å is fully broken. The structural evidence suggests that at alkaline pH the phenoxy core has quinoid character and transforms the lactone ring to an electron rich carboxylate functionality that in turn binds strongly to Ca^2+^. The undecyl arm makes dihedral turns at C_27_ (80°) and C_32_ (72°) that position the diglycolic group exactly on top of the chromophore, sandwiching Ca^2+^ in the middle. Ca^2+^ makes strong interactions with five oxygens; two in the chromophore and three in the receptor, having the following bond distances; 2.47, 2.52, 2.39, 2.50 and 2.52 Å, respectively. The anion-bearing oxygen atom (O_45_) in the receptor has a shorter bond (2.39 Å) with Ca^2+^ compared with the ester carbonyl (O_43_, 2.50 Å) and ether linkage (O_40_, 2.54 Å).

Another case of interest is when **1** interacts with Ca^2+^ at pH neutral. The complex in Figure 1c shows similar characteristics in the xanthene framework as described earlier in Figure 1a. The lactone C_7_-O_22_ bond appears to be intact with a bond distance of 1.55 Å. Ca^2+^ interacts with only the lactone carbonyl (O_25_) in the chromophore with a bond distance of 2.41 Å. The carboxylic (O_45_) and ester (O_43_) carbonyl oxygens give bond distances of 2.52 Å and 2.45 Å, respectively. The ether linkage (O_40_) interacts weakly with Ca^2+^ with a bond distance of 2.61 Å. Ca^2+^ forms a bond angle of 122.4° with the diglycolic carbonyls. Due to restriction in the undecyl arm, the receptor does not make a perfect perpendicular alignment to the lactone ring.

A comparison of key properties in Ca^2+^ complexes with **1** and **1a** is provided in Table 1. In the fully broken C_7_-O_22_ bond in **1a**, the atomic valences decrease to 3.56 and 1.70 for C_7_ and O_22_, respectively, suggesting the formation of ionic centers. Likewise, **1a** is more capable of reducing the charge of Ca^2+^ by more than 5% to 1.79, compared to 1.89 with **1**. The Ca-O_25_ bond in the complex with **1** exhibit greater atomic valence in Ca^2+^ and higher total overlap population. The results suggest more efficient and ionic character in the interaction between Ca^2+^ and **1a** at alkaline pH.

**Figure 2:**
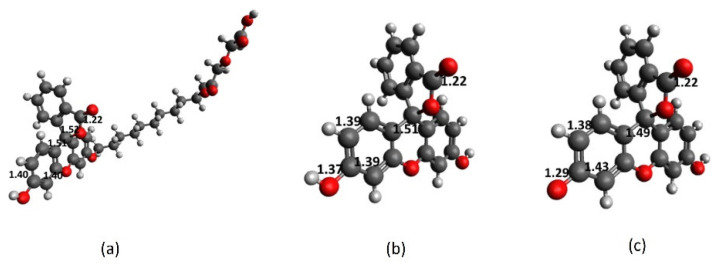
Full-structure optimized geometries in methanol; (**a**) **1**, (**b**) fluorescein, and (**c**) deprotonated fluorescein.

Full structure of fluorescein (Fl) and its deprotonated form in methanol provide insights into the structural effects of fluorescein derivatives such as **1** on its fluorescence activity. The uncharged form exhibits strong aromatic character with a similar C-C bond distance of 1.39 Å in the xanthene core. The C_6_-C_7_ ring linkage and C_7_-O_22_ lactone bond distances are 1.51 and 1.52 Å, respectively (Figure 2b). Deprotonation of phenolic hydrogen (Figure 2c) causes disruption to the xanthene aromaticity; the C_3_-C_4_ elongates to 1.43 Å, indicating a quinoid structure. The formation of the planar 1,4-cyclohexadiene ring system is accompanied by shortening of the C_6_-C_7_ bond to 1.49 Å due to π-overlap within this bond and with its C_1_, C_5_ and C_15_ neighbors.

The optimized geometry of **1** in methanol is obtained by removing Ca^2+^ from the interacting complex (Figure 1c). During optimization, the diglycolic arm rapidly moved away from the chromophore and in the final structure the undecyl arm makes a 45° dihedral angle from the xanthene plane (Figure 2a). The lactone ring is perfectly perpendicular to the xanthene ring system. The uncomplexed **1** exhibits a U-shaped conformation, similar to the non-interacting complex **1**-Ca^2+^ in Figure 1a. The diglycolic receptor is planar and the undecyl arm makes two critical turning points that decisively dictate the final geometry: at C_32_ and O_37_ with 67 and 66° dihedral angles, respectively. Ca^2+^ is bound to **1** through the diglycolic arm that does not interact with the lactone core, as in Figure 1a, by 18.1 kcal mol^−1^. However, Ca^2+^ is bound in Figure 1c by only 17.7 kcal mol^−1^. This means the macrocyclization process that transforms the non-interacting complex in Figure 1a to the interacting one in Figure 1c is disfavored thermodynamically by 0.4 kcal mol^−1^. Considering the uncertainties in the employed level of theory and IEF-PCM model, it is reasonable to assume that the non-interacting and interacting complexes have similar energies.

Significant fluorescence enhancement observed on **1** in the presence of Ca^2+^ could still be rationalized in terms of the formation of the interacting complex in Figure 1c. **1** Could have been pre-organized in a U-shaped conformation seen in Figure 2a. Ca^2+^ could approach **1** via the area between the lactone core and diglycolic receptor. Ca^2+^ is then sandwiched between the two motifs and this process is thermodynamically feasible. The initial formation of the diglycolic-Ca^2+^ complex not in close proximity with the chromophore, but later approaches the lactone moiety is unlikely. Full-structure results reveal key information on cation recognition with **1** and help identify key contributors that could be modeled and studied later in more detail. Eleven methylene units in the undecyl arm gives excellent flexibility for reorganization and optimum interaction with cation and chromophore core. Results from full-structure fluorescein and **1** confirm that anionic substituent breaks the aromaticity of the xanthene core, and two motifs actively participate in Ca^2+^ recognition: diglycolic and lactone.

### 2.2. Chromophore Models and Computational PCA

Chromophore models address key questions regarding the role of fluorescein core in cation recognition: (i) the effect of pH or the role of phenoxy anion on xanthene core, (ii) the versatility of the lactone functionality, (iii) the barrier to the lactone ring opening, (iv) the contribution of the chromophore to chemical selectivity, and (v) the charge transfer to the core and the effect on its structure. Diene lactones **2** and **3** serve as simple models to examine the interaction of chromophore in **1** with cations. Computations with B3LYP/6-311+G(2df,2p) [32] in methanol reveal that cations interact with diene lactone **2** through the carbonyl oxygen (Figure S1b–d).

**Figure 3:**
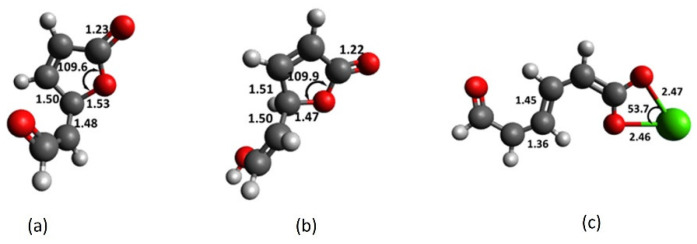
Chromophore models in methanol; (**a**) enolate **3a**, (**b**) enol **3**, and (**c**) complex of **3a** and Ca^2+^ exists in acyclic form.

Using enolate as a model for phenolate, the results in Figure 3a show that at alkaline pH, the lactone C-O bond in **3a** readily breaks and Ca^2+^ interacts with both oxygens in the resulting carboxylate (Figure 3c). Deprotonation of enol hydrogen in **3** lengthens the C_3_-O_7_ bond by 4% and shortens the C_2_-C_3_ bond by more than 1%, by virtue of the negative charge in **3a**. The charge-to-radius ratio of the cation plays a decisive role in the C_3_-O_7_ bond breaking.

**Figure 4:**
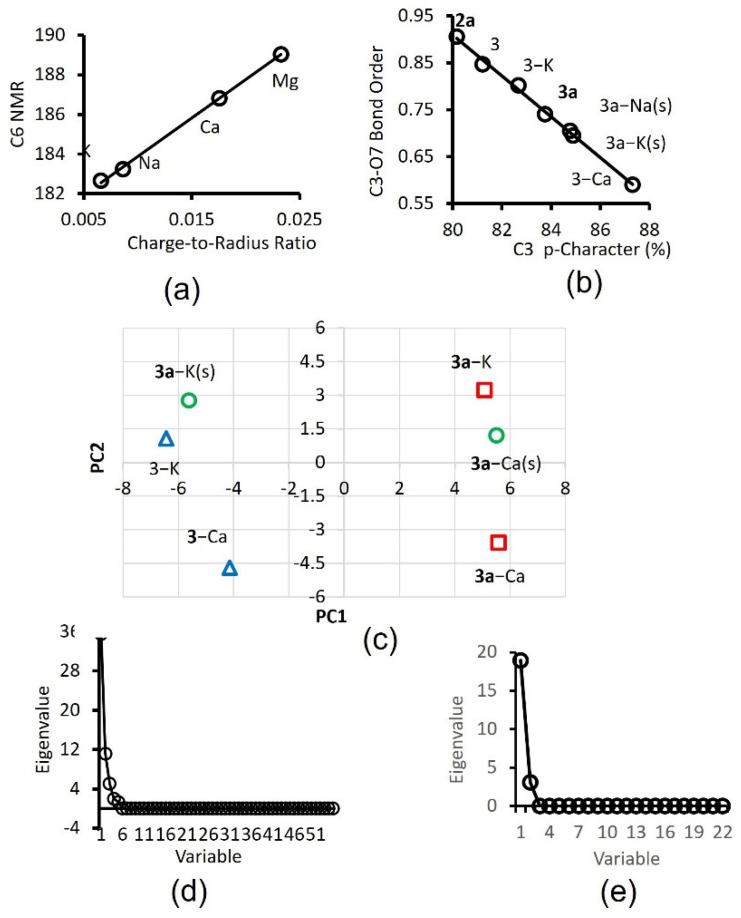
(**a**) C_6_ NMR of **2** vs. cation charge-to-radius ratio in methanol, (**b**) C_3_-O_7_ bond order vs. C_3_ p-character for chromophore models, (**c**) PC2 vs. PC1 score plot, (**d**) computational Scree plot, and (**e**) experimental Scree plot.

Figure 4a shows that the de-shielding of the carbonyl carbon (C_6_) is directly correlated to this ratio. Another key observation is the increase in the C_3_ p-character that accompanies the C_3_-O_7_ bond breaking. It indicates the formation of a double bond and π-network (Figure 4b). Cation transfers its charge mainly to the carbonyl carbon (C_6_), as can be observed in the de-shielding of this nucleus. Chromophore-cation binding strength can be rationalized in terms of stabilization of the HOMO of the chromophore (Figure S2a) [33]. The cation-catalyzed ring opening of the lactone functionality occurs readily and gives rise to the participation of the chromophore (as carboxylate motif) in cation recognition, as described later in Section 2.7.

The structural and electronic properties of chromophore models, including those in the supplementary information, are utilized as variables in multivariate analyses of these systems. Over fifty variables including bond distance, bond angle, dihedral angle, NBO charge [34], AOMix [35] atomic valence, AOMix total overlap population, NMR GIAO shielding coefficient [36] and bond dissociation energy are used as variables in PCA. Variables from two chromophore systems, enol **3** and enolate **3a** were the main sources due to economic reasons. Data from the gas phase are compared with those in methanol medium (depicted in Figure 4c as (s)). Determination of two cationic plant macronutrient, Ca^2+^ and K^+^, is the main focus of this study (Figure 4c).

The scree plot in Figure 4d shows the successful transformation of the variance in the data to three important principal components, PC1, PC2 and PC3, having eigenvalues of 35.5, 11.2 and 5.0, respectively [37]. Figure 4c displays a score plot of PC2 versus PC1, showing three pairs of chromophore-cation models [38]. There are six points or three Ca^2+^-versus-K^+^ pairs of interest on the score plot. The scores preferably are far away from the origin and separated into four distinct quadrons, hereinafter referred to using the following convention: top-left (Q1), top-right (Q2), bottom-right (Q3) and bottom-left (Q4). The first case involves the separation of **3a**-Ca^2+^ from **3a**-K^+^ in a vacuum. The two points (red squares) are very well separated vertically on the right into Q3 and Q2 quadrons, respectively.

The second scenario is more important to the practical application since it involves the separation of this pair in the actually used solvent (i.e., methanol denoted by (s)). The two points (green circles) are even better separated into Q2 and Q1 quadrons, respectively. The pair with enol **3** chromophore (blue triangles), **3**-Ca^2+^ and **3**-K^+^, is also well separated vertically on the left into Q4 and Q1 quadrons, in that order. The results suggest that the chromophore-cation systems afford distinct clusters and variations in the computational molecular properties give rise to good cluster separation. Score plots of PC3 versus PC1 and PC3 versus PC2 have also been analyzed and confirmed most of the extracted information above, albeit with less separations in certain pairs.

A scree plot from actual experimental data, employing **1** and fluorescein as fluorophores and with Ca^2+^ and K^+^ macronutrients, is provided in Figure 4e for comparison. The PCA utilized 22 emission intensities from 500 to 600 nm as variables. Diene lactones have successfully been employed as models in elucidating the nature of interaction between cations and the xanthene core and the effect of anionic substituent on lactone structure, as well as in providing molecular properties as variables in PCA. Our results show that ene- and diene lactones are responsive towards cation, and computational data can be employed in PCA to evaluate potential motifs for cation recognition. Facile ring opening at alkaline pH transforms the lactone moiety to carboxylate functionality that enhances selectivity towards Ca^2+^.

### 2.3. Diglycolic Ca^2+^ Receptor Models

**Figure 5:**
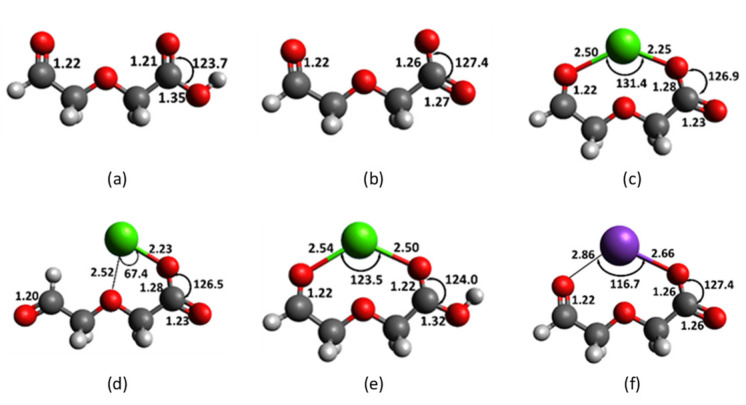
Optimized geometries of **4** and **4a** in methanol; (**a**) **4**, (**b**) **4a**, (**c**) complex of **4a** and Ca^2+^, (**d**) complex of **4a** and Ca^2+^ with 2 oxygens, (**e**) complex of **4** and Ca^2+^, and (**f**) complex of **4a** and K^+^.

Computation on diglycolic models seeks to understand the following: (i) the origin of Ca^2+^ selectivity with this receptor, (ii) the participation of various types of oxygen atoms, and (iii) the effect of negative charge. This work focuses on two forms of diglycolic moiety as receptor for Ca^2+^; diglycolic acid **4** and diglycolate anion **4a** (Figure 5). In the absence of cation, both **4** and **4a** have been optimized as planar structures. In **4** the carboxylic hydrogen on O_8_ is in cisoid conformation with O_7_. For simplicity and economic reasons hydrogen is used as a substitute for the undecyl arm in **1**. The two carbonyl C-O bond distances in 4 are similar, with a baseline length of 1.22 Å at the aldehyde terminal. The carboxylic C5-O8 bond is 1.35 Å, whereas, when deprotonated in **4a**, the carboxylate C-O bonds have a common bond distance of 1.26 Å due to resonance. The distances between the two carbonyl oxygens in **4** and **4a** are 4.78 and 4.33 Å, respectively.

The distance between the carbonyl oxygen (O_6_) at the aldehyde terminal and the ether linkage (O_3_) is 2.74 Å. Cation recognition by **4** or **4a** most probably involves three oxygens in the diglycolic receptor. Ca^2+^, Mg^2+^ and K^+^ having Shannon ionic diameter [39] of 2.28, 1.72 and 3.04 Å, respectively, position itself in the middle, and make distinct cation-O bond distances and O-cation-O bond angles. The size of the cation and its charge-to-radius ratio are deciding factors that influence how best the cation fits into the cavity between the three oxygens. The **4a**-Ca^2+^ complex in Figure 5c has bond distances of 2.50, 2.41 and 2.25 Å, each between Ca^2+^ and O_6_, O_3_ and O_7_, respectively. The carboxylate anion exhibits a short Ca-O_8_ bond of 2.25 Å. Ca^2+^ makes a 131.4° bond angle with the two carbonyls. In Figure 5d, O_6_ is not available for interaction and Ca^2+^ binds with O_3_ and O_7_ to give 2.52 and 2.23 Å bond distances, respectively, making a narrow O_3_-Ca-O_7_ angle of 67.4°.

Bonding with only two instead of three oxygens makes the **4a**-Ca^2+^ complex less stable by 9.1 kcal mol^−1^. In contrast to the deprotonated form, the **4**-Ca^2+^ complex in Figure 5e forms noticeably longer bond distances of 2.54 and 2.50 Å with O_6_ and O_7_, respectively. The O_3_-Ca-O_7_ angle was significantly reduced to 123.5°. The Ca-O_3_ bond distance of 4.78 Å indicates negligible interaction between Ca^2+^ and the ether linkage (O_3_) in the protonated receptor. Likewise, the long Ca-O bond distances of 2.86 and 2.66 Å with O_6_ and O_7_ in **4a**-K^+^ indicate even weaker interaction with alkaline metals (Figure 5f).

Diglycolic dialdehyde **4b** is a further simplified model for the Ca^2+^ receptor. This model is useful in examining the uncharged receptor, having identical carbonyl motifs. Binding energies in Table 2 indicate that Ca^2+^ is bound to dialdehyde **4b** in methanol by 11.6 kcal mol^−1^. The **4b**-Ca^2+^ complex is capable of further binding with diene-lactone **2** with additional 5.6 kcal mol^−1^ of binding strength. In striking contrast to the full-structure results, in separate fragment models, initial formation of the diglycolic-cation complex followed by the additional interaction of this complex with the chromophore lactone is thermodynamically feasible and is exothermic by more than 5 kcal mol^−1^. Difficulty in macrocyclization in Fl-DGA-Ca^2+^ complex involving initial formation of diglycolic-Ca^2+^ complex could be explained using the entropy argument [40].

The interacting and non-interacting **1**-Ca^2+^ complexes have similar energies and thus additional interaction with the lactone group does not result in further stabilization. The undecyl arm could not have interacted with Ca^2+^ in an initial step. Likewise, initial binding between Ca^2+^ and the lactone motif in 1 is also unlikely, although the fragmented models suggest that the complex is bound by 6.5 kcal mol^−1^. In the carboxylate models (**4a**), Ca^2+^ binds to the anionic form by additional 15.4 kcal mol^−1^ compared with the binding energy with the uncharged form (**4**). A similar trend is true with K^+^ but the alkaline metal is only bound by 7.2 kcal mol^−1^ in methanol.

With ene-lactone **2a**, alkaline earth and alkaline metal cations exhibit the following trend in binding energies: Mg^2+^ > Ca^2+^ > K^+^~Na^+^, and, likewise, with diglycolate **4a** the three cations of interest afford the following order: Mg^2+^ > Ca^2+^ > K^+^. Binding strengths alone do not account for Ca^2+^ selectivity. The results, nonetheless, highlight the advantage of involving three oxygens instead of just two, and the importance of ionization with the diglycolic motif. The computational results show that all diglycolic-cation complexes are planar and attempts in locating complexes involving four oxygens have been unsuccessful. In the hypothetical four-oxygen interaction, the complex would have been bent. Fragmented models confirm that in a stepwise mechanism involving the initial formation of receptor-cation complex, the subsequent interaction with lactone motif is exothermic by 5 kcal mol^−1^. A long distance between the two carbonyl oxygens in diglycolic receptor, fortuitously in a 1, 5 position, and the ionizable nature of carboxylic functionality, tend to favor large and polarizable divalent cation such as Ca^2+^.

### 2.4. Interaction of Receptor and Chromophore Fragments with Cation

**Figure 6:**
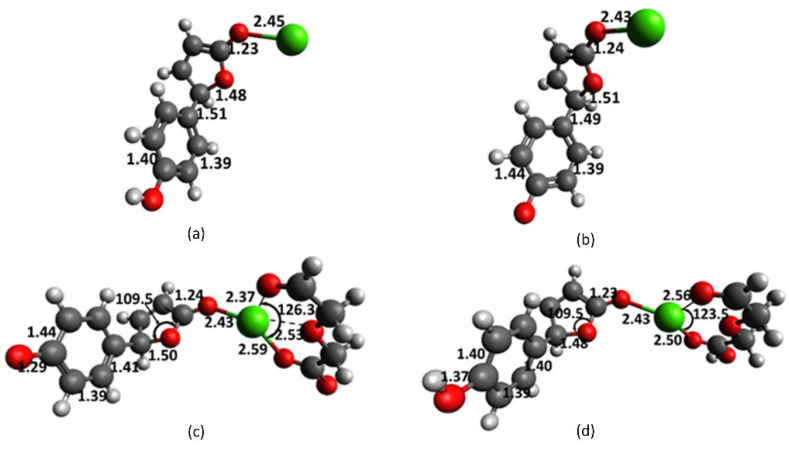
Optimized geometries of complexes with **5** and **5a** in methanol; (**a**) complex of **5** and Ca^2+^, (**b**) complex of **5a** and Ca^2+^, (**c**) **5a**-**4a**-Ca^2+^ complex, and (**d**) **5**-**4**-Ca^2+^ complex.

In Section 2.2, vinyl, enol and enolate were utilized as simple models for the phenol and phenoxy core. Chromophore **5** and **5a** utilize full phenol and phenolate structures and thus serve as better models to examine the interactions of key contributors in **1**, referred to as fragments, with cations. This section seeks to understand the structure of chromophore-receptor-cation complexes, utilizing fully functional models, albeit not bonded together via an extended arm. In methanol medium, **5** interacts with Ca^2+^, resulting in the elongation of the lactone C_7_-O_11_ and carbonyl C_10_-O_13_ bonds to 1.48 and 1.23 Å, respectively (Figure 6a). The aromatic C-C and the ring-linkage C_4_-C_7_ bonds remain practically unchanged.

Deprotonation of phenolic hydrogen causes the C-C bonds in the aromatic ring to be dissimilar, wherein the C_1_-C_2_ bond significantly lengthens to 1.44 Å. The lactone C_7_-O_11_ bond lengthens further to 1.51 Å, and the ring-linkage (C_4_-C_7_) and Ca-O_13_ bonds shrink to 1.49 and 2.43 Å, respectively. These results suggest that as the C_7_-O_11_ bond breaks, a quinoid π-network that extends to the lactone ring forms and is characterized by C_4_-C_7_, having a double bond character. The interaction of 1 or its deprotonated form **1a** and Ca^2+^ presumably involves a pre-organized structure that allows the diglycolic moiety and the lactone group, perpendicular to the xanthene plane, to simultaneously interact with the incoming cation. Therefore, cation-catalyzed lactone ring openings of **1** and **1a** do not involve the initial formation of the chromophore-Ca^2+^ complexes modeled in Figure 6a,b.

The result in Figure 6c suggests that the interaction of the receptor-Ca^2+^ complex with the chromophore core affords a stable larger complex. However, in striking contrast to **1a**-Ca^2+^, this does not lead to a greater extent of lactone C_7_-O_11_ bond breaking. The Ca-O_13_ bond distance remains at 2.43 Å and the rest of the features in the chromophore are not noticeably different from those in Figure 6b. A shorter C_7_-O_11_ bond distance of 1.50 Å in **5a**-**4a**-Ca^2+^, compared with that in **5a**-Ca^2+^, suggests an even lesser extent of bond breaking in the former. It is plausible that Ca^2+^ first binds with **5a** through the electron-rich O_11_, instead of carbonyl O_13_ because the C_7_-O_11_ bond is partially broken by virtue of the anionic character. Likewise, similar initial interaction could happen in dianion **1a**, involving the anionic chromophore, albeit having the ionized diglycolic arm in close proximity.

It is noteworthy that the Ca-O_13_ bond distance involving uncharged phenol core **5** and diglycolic acid receptor **4** in Figure 6d remains at 2.43 Å (as in Figure 6b,c). The rest of the chromophore features in the **5**-**4**-Ca^2+^ complexes are comparable to those in **5**-Ca^2+^. If it is viewed that the **5**-Ca^2+^ complex forms first, receptor **4** approaches the complex, perpendicular to the lactone ring, and as the larger complex forms the Ca-O_13_ bond shrinks from 2.45 to 2.43 Å. The **5a**-**4a**-K^+^ complex in Figure S4c could be viewed as having a phenoxy core that does not exhibit any structural change due to complexation. The **4a**-K^+^ complex could form first and interact with **5a**, or vice versa, **5a**-K^+^ could approach **4a**, giving rise to the Ca-O_13_ bond distance of 2.75 Å but leaves the entire structure of **5a** practically unchanged.

Full-structure computations of interacting complexes involving **1** and **1a** suggest that when the chromophore and the receptor components are bonded together via an extended arm, the two parts could, in the presence of cation, approach each other in near perfect perpendicular fashion. The results in Table 3 show that the breaking of the lactone C_7_-O_11_ bond in **5a**-**4a**-Ca^2+^ is accompanied with a significant decrease in bond order and total overlap population in this bond. The reduction in C_4_ chemical shift indicates shielding due to the accumulation of electron density surrounding this nucleus, or, alternatively, because of the loss of the aromatic ring current. Likewise, the increase in C_7_ chemical shift suggests the increased vinylic character of this carbon and participation in the newly formed π-network. The C_4_ does not exhibit a noticeable difference in the p-character in the bonding orbital. It remains at 70% or equivalent to the sp^2.3^ hybrid orbital since it involves changing from aromatic carbon to a vinylic one. The dianion form reduces more positive charge from Ca^2+^, but this is not accompanied by additional positive charge buildup at the carbonyl C_10_. Fragmented models further support the influence of negative charge on lactone motif and its contribution to cation recognition. However, a comparison of complexes with dianions, **1a**-Ca^2+^ and **5a**-**4a**-Ca^2+^, raised an important question: why does the lactone C-O bond break completely with full-structure **1a** but only minimally with fragments of **5a** and **4a**? We propose that this arises from the rigid structure of **1a**, wherein the diglycolate motif approaches the chromophore at a right angle to the lactone ring, achieving five optimized Ca-O bond distances by first ripping the lactone C-O bond.

### 2.5. Optical Response Characterization

In optical characterization, we investigate the fluorescence response of **1** with three cationic macronutrients: Ca^2+^, K^+^ and Mg^2+^. Selectivity of **1** with cations is compared to those with other fluorophores: unsubstituted fluorescein, lipophilic derivative **7** and rhodamine-B. Capability for simultaneous determination of cations and resolving signal at sub mm levels are assessed from the quality of separation in the score plots. The optical sensor utility of **1** was explored by dissolving the fluorophore in methanol to form yellowish solutions that are stable at different concentrations (3.2, 1.0, 0.3 and 0.1 mM) for over two weeks. As can be seen from Table 4, the maximum fluorescence intensity (I_m_/I_0_) of **1** was observed when 1 mM solution of the fluorophore and 0.1 mM of metal cation were employed. Maximum emission peaks at wavelength of 544 ± 3 nm for all tested concentrations of **1** was observed due to high fluorescence quantum yields. Low peak intensities were apparently observed at higher concentrations (3.2 mM) due to a quenching effect, as has been reported by many studies previously. For example, the quenching effect of chloride on quinine fluorescence activity has been well documented [41].

**Figure 7:**
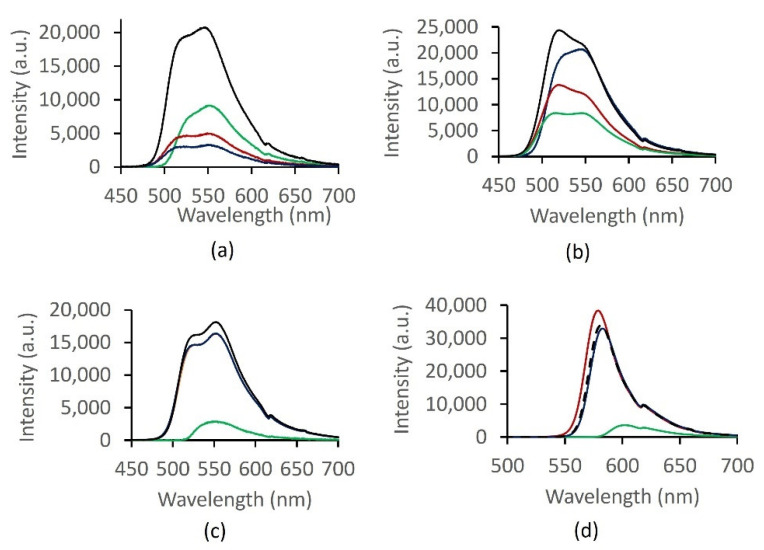
Emission intensities with Ca^2+^, K^+^ and Mg^2+^; (**a**) **1**, (**b**) Fl, (**c**) Fl-OH and (**d**) Rh. Color: black (Ca^2+^), red (K^+^), blue (Mg^2+^) and green (fluorophore).

Fluorescence spectroscopic tests were also carried out by the addition of chloride salts of three cations of interest in precision agriculture; Ca^2+^, Mg^2+^ and K^+^ (0.1 mM), into four 1 mM chromophores solution (fluorescein (Fl), lipophilic alcohol fluorescein derivative (Fl-OH), Fl-DGA and rhodamine-B (Rh-B)) and the effect of metal ions on fluorescence intensity of each chromophore system was examined. Figure 7a shows that Mg^2+^ and K^+^ give rise to quenching effect on the fluorescence intensity of 0.1 mM **1**. On the contrary, significant fluorescence enhancements were observed with Ca^2+^, yielding the highest enhancement at 1 mM concentration of **1**. Due to high charge-to-radius ratio and strong solvation of the two alkaline earth metals, comparable optical responses were expected with Mg^2+^ and Ca^2+^. Quite the contrary, our results suggest that **1** exhibits the ability to be selective towards Ca^2+^ and discriminate against Mg^2+^ and K^+^. Selectivity towards Ca2+ has been reported previously with tetracarboxylate chelators [42].

### 2.6. PCA of Optical Responses

Fluorescence emission intensities of 1 × 10^−5^, 1 × 10^−4^ and 1 × 10^−3^ M (subsequently referred to as 0.01, 0.1 and 1 mM or alternatively referred to in the legend as the abbreviated logarithmic form −5, −4 and −3 M, respectively) of Ca^2+^ and K^+^ with 1 × 10^−3^ M of fluorophore in methanol were used in multivariate analyses. Unsubstituted fluorescein (Fl), Fl-DGA, lipophilic alcohol fluorescein derivative (Fl-OH) and rhodamine-B (Rh-B) at 1 mM concentration were evaluated for to determine calcium using the PCA method. Based on its fluorescence emission characteristics, optical responses between 500 and 600 nm are suitable for Fl, Fl-DGA and Fl-OH.

**Figure 8:**
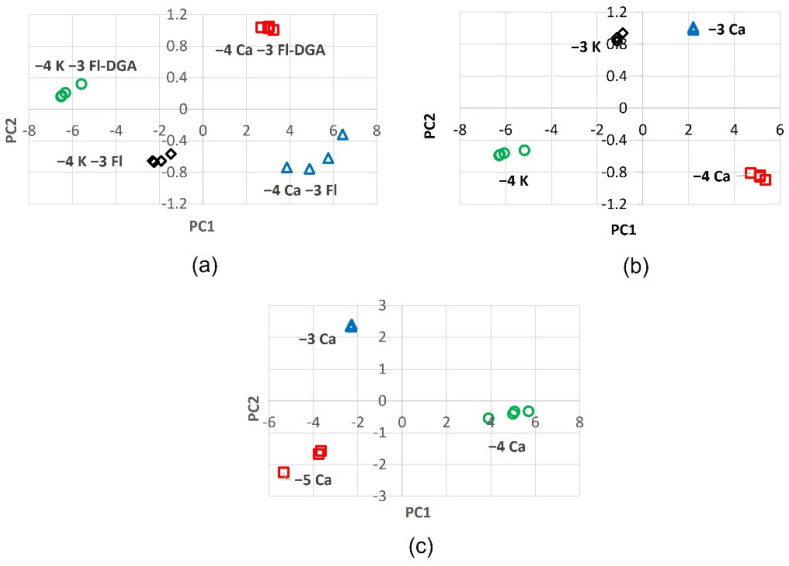
Score plots; (**a**) 0.1 mM K^+^ and 0.1 mM Ca^2+^ in 1 mM **1**, (**b**) 0.1 mM K^+^, 0.1 mM Ca^2+^, 1 mM K^+^, 1 mM Ca^2+^ in 1 mM **1**, and (**c**) 0.01, 0.1 and 1 mM Ca^2+^ in 1 mM **1**.

Between twenty to sixty responses were utilized as variables in the principal component analysis. Successful extraction of principal components is characterized by a normal scree plot (Figure 4e) that can be interpreted as having sufficient variance in the data and the information reduced to a few dimensions having the highest eigenvalues. More importantly, in Ca^2+^ determination, the first two principal components should produce a score plot (PC2 vs. PC1) that exhibits distinct and well separated clusters. The results in Figure 8 suggest that over twenty experimental variables (22 emission intensities are used) afford decent principal components and an increase in the number of variables does not necessarily improve cluster separation. Since the PCA procedure allows only a comparison of common variables, rhodamine that exhibits a narrow emission window of 550–600 nm could not be employed in subsequent measurements and multivariate analyses. As in Section 2.2, the same naming convention is adopted: top-left (Q1), top-right (Q2), bottom-right (Q3), and bottom-left (Q4).

The first case of interest is the determination of 1 × 10^−4^ M (0.1 mM) of Ca^2+^ and its separation from 0.1 mM potassium (Figure 8a). Moreover, it is important to compare the score plot of Fl-DGA with that of the parent fluorescein compound (Fl). It is noteworthy that the four possible clusters, from four sets of samples, afford distinct clusters in dedicated quadrons. Fl-DGA separates 0.1 mM Ca^2+^ and 0.1 mM K^+^ into the Q2 and Q1 quadrons, respectively. Both cations exhibit tight clustering near (3,1) and (−6,0.2) coordinates, respectively. Fl separates the two cations at 0.1 mM, less effectively into the Q3 and Q4 quadrons. The 0.1 mM Ca^2+^ scores in Fl fluorophore are also poorly clustered, horizontally spread from 4 to 6 along the PC1 axis. These results confirm the photophysical observations that indicate improved responses with Fl-DGA and Ca^2+^.

The second scenario further examines the performance of Fl-DGA with different concentrations of Ca^2+^ and K^+^ at millimolar range. Results in Figure 8b show that Fl-DGA is capable of separating Ca^2+^ and K^+^ much more efficiently at 0.1 mM. The 0.1 mM Ca^2+^ and K^+^ are separated into tight clusters at the Q3 and Q4 quadrons, respectively. The 1 mM cations are located at the top quadrons, albeit only minimally separated. Despite the lack of separation, the scores of 1 mM cations appear to cluster more tightly. These results suggest that Fl-DGA has superior performance at sub-millimolar ranges.

Trace-level detection is of interest in water quality [43] and environmental monitoring. The optical responses and cluster separation at 0.01 mM indicate capability at sub-millimolar ranges. Figure 8c shows the good separation of 0.01, 0.1 and 1 mM Ca^2+^ by Fl-DGA. The good separation between 0.1 and 1 mM Ca^2+^ is retained by having the scores at the Q3 and Q1 quadrons, respectively. Moreover, similar to the observation in Figure 8b, a higher concentration of Ca^2+^ affords superior clustering. More importantly, 0.01 mM Ca^2+^, at the middle of Q4 quadron, is well separated from the rest of the scores, albeit less tightly clustered. The above experimental evidence confirms that fluorescent receptor **1** is selective towards Ca^2+^. The excellent separation of tightly populated clusters in the score plots proves that the probe can be employed for the simultaneous measurement of cationic macronutrients and detection at sub-millimolar levels.

### 2.7. Mechanistic Insights and Rationale for Calcium Selectivity

Computational bond dissociation energies in Table 2 are consistent in predicting the following preferential binding interactions between chromophore and receptor models with three cations of interest: Mg^2+^ > Ca^2+^ > K^+^. With ene-lactone **2a**, Mg^2+^ is twice as strongly bound compared with Ca^2+^, and K^+^ is bound by only 4.3 kcal mol^−1^ and can be discriminated against. Likewise, with diglycolate anion **4a**, Mg^2+^ is more strongly bound by two and a half times compared with Ca^2+^, while the interaction with K^+^ is only half that of Ca^2+^. On the contrary, optical responses with Fl-DGA (**1**) exhibit quite the opposite trend: Ca^2+^ >> K^+^~Mg^2+^. Furthermore, a decisive role of the charge-to-radius ratio of cations on the nature of lactone carbon-oxygen bond and other physical and electronic properties is consistent with all computational findings. Factors that contribute to the observed selectivity and insights into the roles of anion, the lactone motif, solvation and diglycolic receptor will be discussed further.

**Figure 9:**
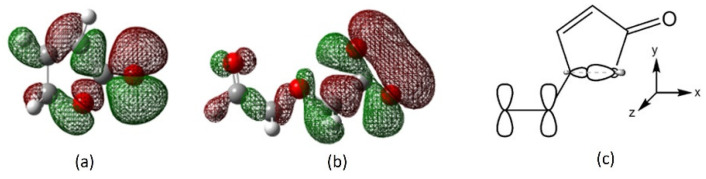
Frontier orbitals of **2a** and **4a**; (**a**) HOMO of **2a**, (**b**) HOMO-1 of **4a**, and (**c**) orbitals involved in C-O bond breaking.

In the absence of solvation, cations bind with both oxygens in the lactone moiety. Cation approaches **2a** from the same plane of the molecule and in the middle between ring oxygen (O_5_) and carbonyl oxygen (O_6_). The HOMO of **2a** has a large in-plane lobe for frontal attack that covers this area (Figure 9a). Alternatively, the chromophore could approach a receptor-cation complex through the LUMO+1 which provides favorable frontal interaction (Figure S3b). Calcium interacts with the carbonyl and ether oxygens in **4** through the HOMO or HOMO-1 (Figure S4d,e). In **4a**, the HOMO-1 provides favorable interaction with both carboxylate oxygens (Figure 9b). Resonance contributor in the lactone ring causes the carbonyl oxygen to be more negatively charged and is more strongly bound to cation than the ring oxygen is. In the IEF-PCM model, solvent molecules hinder interaction between cation and the ring oxygen, leaving carbonyl-cation interaction as the main factor in cation recognition.

**Figure 10:**
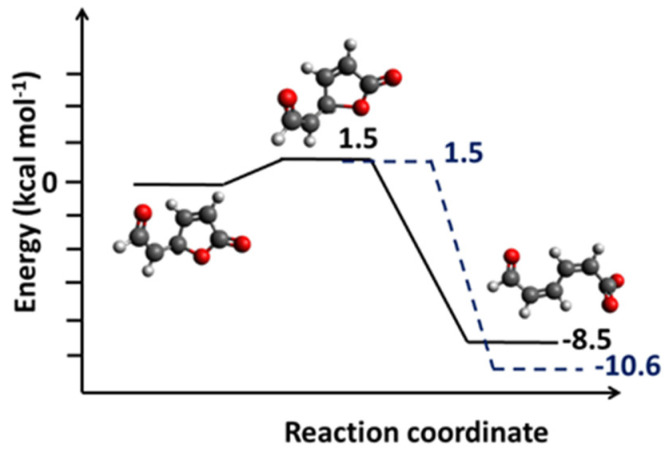
Barrier of C-O bond breaking in enolate **3a** chromophore model. Solid line is profile in vacuum and dotted line in methanol medium.

By virtue of the negative charge on C_1_, the C_3_-O_7_ bond in **3a** lengthens to 1.53 Å (compared with 1.47 Å in **3**). While interaction with Ca^2+^ leaves the lactone ring in enol **3** intact, the cation catalyzes the ring opening of enolate **3a** (Figure 3c). The C_2_-C_3_ bond shrinks to 1.36Å, suggesting a double-bond character. Likewise, shortening of the C_3_-C_4_ bond to 1.45 Å indicates the formation of the π-network (Figure 9c). Calcium interacts strongly with both carboxylate oxygens, affording a Ca-O bond distance of 2.47 Å and O7-Ca-O9 bond angle of 53.7° (Figure 3c). The results imply that the presence of a negatively charged substituent changes the chemistry of the lactone ring in fluorescein derivatives and influences its role in cation recognition. Cation catalyzes the facile ring opening of enolate **3a** (Figure 10) with an early transition state and a low activation barrier of just 1.5 kcal mol^−1^. The transition structure exhibits new single and double C-C bonds, but the carboxylate functionality has not fully formed, and the geometry has not flattened. The transition structure is further characterized by having one imaginary frequency at −304 cm^−1^, C_3_-O_7_ bond distance of 1.83 Å and IRC paths that connect to enolate 3a and the ring-opened product [44]. In methanol, the lactone ring opening process is more exothermic by 2 kcal mol^−1^ but has the same activation barrier (Figure S5).

Computational binding energies from full structures and simple models consistently predict greater binding strengths with Mg^2+^, whereas experimental optical responses suggest selectivity towards Ca^2+^. Apparently, computational bond dissociation energies alone cannot explain the experimental observation. The fluorescein core and diglycolic receptor presumably contribute to selectivity towards Ca^2+^. Useful insights could be gained from the well documented selectivity of ionomycin towards Ca^2+^ [45]. The nature of Ca^2+^ itself most probably is key to the observed selectivity with both **1** and ionomycin.

Although binding energies alone could not account for Ca^2+^ selectivity, computational results are consistent in highlighting the importance of the charge-to-radius ratio of the cations. Charge density per unit area on Ca^2+^ is much smaller than that on Mg^2+^. Therefore, fluorophore, having flexible ionizable arms, can stabilize Ca^2+^ much more effectively through ion-pair mechanism and discriminate against other cations. On the contrary, K^+^ and other monovalent cations favor neutral recognition molecules and can be effectively determined through the size exclusion mechanism [46]. Magnesium recognition is more elusive [47] but computational natural bonding orbitals, atomic valence and charge analysis suggest that Mg^2+^ selectivity most probably arises from effective stabilization of the divalent cation that exhibits high charge density and strong tendency towards covalent interaction. At alkaline pH, the fluorescein core and diglycolate moieties in 1a function as two arms that can freely reorganize to effectively stabilize Ca^2+^. Rather fortuitously, the entire fluorescein structure acts in favor of Ca^2+^ recognition; it serves as the second arm that provides stability to Ca^2+^ by virtue of its carboxylate motif.

Carbonyls, polyethers, peptides and heterocycles have successfully been employed as motifs in cation recognition. The effective use of dicarbonyls as cation receptors is of interest in this study. Diglycolic acid **4** can be viewed as a 1,5-dicarbonyl receptor, having an ether linkage and ionizable carboxylic moiety. The long distance between the two carbonyl groups apparently favors interaction with a large and polarizable divalent cation such as Ca^2+^. Moreover, the ether linkage participates in cation binding, and the ionizable carboxylic functionality plays a key role in Ca^2+^ recognition. While the carboxylic moiety is also present in ionomycin, the dicarbonyl presents in the 1,3-position, making the distance between the two carbonyls much shorter and the α-protons significantly acidic for facile ionization. Instead of an ether linkage, ionomycin has two tetrahydrofuran rings that interact with Ca^2+^.

What then is the origin of Ca^2+^ selectivity in ionomycin and **1**? There are two main reasons that determine the sensor signal: (i) the cation-receptor binding strength, and (ii) the probability of the cation being in contact with the recognition molecule. While binding energies predict stronger interaction with Mg^2+^, optical response confirms the selectivity towards Ca^2+^. Logically, something must have prevented Mg^2+^ from coming into close proximity with the receptor. The medium that surrounds Mg^2+^ is the most plausible agent that slows down its mobility due to its high charge density. Tight solvent cage encapsulates Mg^2+^ and hinders its movement. The Hofmeister effect, which favors large monovalent cation, invokes a similar argument [48]. Succinctly, **1** filters out Mg^2+^ due to the Hofmeister effect and discriminates against K^+^ via the ion-pair mechanism.

## 3. Conclusions

The simulation of full-structure **1** and **1a** in methanol provides insights into the observed fluorescent enhancement with Ca^2+^. Selectivity towards Ca^2+^ and discrimination against Mg^2+^ and K^+^ are attributed to diglycolic and lactone motifs. The extent of the lactone C-O bond breaking is indicative of fluorescence activity. As the C-O bond breaks, the xanthene ring loses aromaticity and exhibits dissimilar C-C bonds. The C-O bond breaking, enhanced by the presence of anionic substituent, transforms the lactone motif to a carboxylate functionality that actively participates in Ca^2+^ recognition. Diene lactone models elucidate the nature of chromophore-cation interaction and provides molecular properties as variables in multivariate analysis. PCA score plots afford distinct and well-separated clusters and prove that computational approaches can be used to evaluate potential chromophores and receptors in the determination of cations for sensor array applications. Fragment models support the full-structure findings that the anionic substituent causes structural changes but does not reproduce the full extent of lactone C-O bond breaking observed in full-structure **1a**-Ca^2+^. Rigid structure in 1a is attributed to the complete breaking of the C-O bond.

Fluorescein derivative **1** was prepared by functionalization of the parent molecule with undecyl arm having a diglycolic moiety at the terminal. Binding energies suggest similar energies for **1**-Ca^2+^, regardless of additional interaction with the lactone motif. In a pre-organized U-shaped **1**, Ca^2+^ can simultaneously interact with the lactone and diglycolic motifs. Emission intensities of **1** with mM and sub mm concentrations of Ca^2+^ and K^+^ yield score plots with distinct clusters and are thus suitable for simultaneous detection of these macronutrients in precision agriculture application. The size and charge density of Ca^2+^ are key factors in the rational design of its receptor. Insights from ionomycin suggest the importance of ionizable motifs in Ca^2+^ recognition. At an alkaline pH, deprotonation exposes anionic sites that stabilize the large and polarizable divalent cation via an ion-pair mechanism. Despite stronger binding interaction with the diglycolic receptor, Mg^2+^ is excluded due to its high charge density, poor mobility in polar medium and preference towards covalent bonding.

We have adopted the following methodology in the rational design of a cation receptor that could be generalized for the design of other fluorescent probes: (i) compute full-structure geometries of proposed molecule, (ii) identify key contributing components to a desired recognition, (iii) examine the nature of simple models, (iv) compare geometries of full and fragmented complexes, (v) determine optical responses and detection limits, (vi) account for observed selectivity, (vii) assess multiple-ion capability using PCA, and (viii) generalize the method, evaluate new motifs and target other applications. As evidenced by this report, the methodology involves the above activities, but not necessarily in any particular order. Key activities are most likely performed in parallel, and it is not uncommon that the end triggers the beginning of a new system.

Our findings show that conveniently prepared fluorescent molecules could be exploited in selective Ca^2+^ determination. In the sensor array and multivariate approach, several cations at mM and sub mm levels can be simultaneously detected for a wide range of applications, including precision agriculture and water quality. The combined computational and experimental approach has proven to be beneficial in the rational design of cation receptors. The approach can be extended to other cations of interest in the following areas: trace-level heavy metal detection, determination of ionic mixtures, precision measurement of anionic macronutrients [49], and rational design of Mg^2+^ selective fluorescent probe. Fluorescent probes provide an advantageous chemical sensing platform that does not depend on reference electrodes and thus is less prone to errors. It is anticipated that fluorescein, rhodamine and eosin derivatives will continue to play a dominant role in optical sensors. The sensitivity of such an optical sensor could also be improved by incorporating surface plasmon-coupled directional emission as the detector, which could offer new physicochemical insights into the interaction mechanism for such chromophores with its target [50,51]. We are motivated to explore the utility of other versatile motifs such as peptides via facile functionalization of xanthene chromophores.

## 4. Experimental Section

### 4.1. Materials and Methods

The chemicals and metal ions used: fluorescein, 4-dimethylaminopyridine (DMAP), 11-bromo-1-undecanol and diglycolic anhydride, were purchased from Sigma-Aldrich (Burlington, MA, USA) and Merck (Darmstadt, Germany) and used without further purification. Acetonitrile, ethanol, ethyl acetate, n-hexane, chloroform and anhydrous N,N’ dimethyl formamide (Sigma Aldrich) were all analytical grade and used without further purification. Deionized distilled water and methanol with HPLC grade, available from Merck (Darmstadt, Germany), were used as the solvent. Acetonitrile and triethylamine (Sigma Aldrich) were freshly distilled over barium oxide before use.

The melting points of samples were measured using a U Met-temp II Laboratory Devices USA. The FTIR spectra were recorded between 4000 and 400 cm^−1^ as attenuated total reflectance (ATR) on a Perkin Elmer Spectrum 400 FTIR spectrometer. ^1^H and ^13^C NMR were recorded on a Bruker Advance 400 spectrometer at 400 and 100 MHz, respectively, using tetramethylsilane (TMS) as the internal standard. The CHN elemental analysis of fluorescein derivatives were determined using a Perkin Elmer CHNS/O 2400 Series II. Fluorescence spectra were obtained using a Spectrometer Avantes 2048-L (Avantes, Netherlands) equipped with an AvaLight-XE LED light source. The spectrometer and the light source were both kept at a constant temperature of 28 °C ± 0.8 °C.

### 4.2. Synthesis

**Scheme 2:**
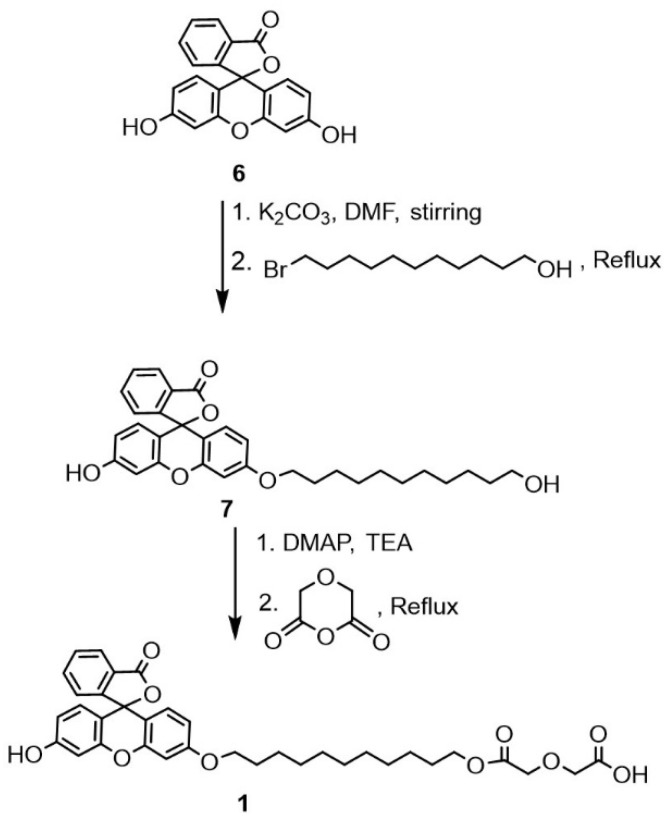
Synthesis of Fl-DGA.

We adopt a three-step approach in preparing fluorescent cation receptors, using unsubstituted fluorescein as a starting material: (i) attach extended arm, (ii) functionalize arm with main motif, and (iii) convert ionizable terminals to esters or amides. Preparation of Fl-DGA, via lipophilic alcohol **7** and without further derivation of the resulting acid, is provided in the following. To a mixture of fluorescein **6** (1 g, 0.3 mmol) and potassium carbonate (0.9 g, 0.6 mmol) in dry DMF, 11-bromo-1-undecanol (0.6 mmol) was added in a dropwise manner and the mixture was refluxed overnight under an inert atmosphere (Scheme 2). The progress of the reaction was monitored by TLC, which indicated formation on a new product and completion of reaction after 18 h. The mixture was rotary evaporated and extracted with ethyl acetate, followed by 20 mL of distilled water (5 times) and the product (**7**) was purified by column chromatography with 2:1 ratio of ethyl acetate/*n*-hexane eluent.

**7** (3′-hydroxy-6′-((11-hydroxyundecyl)oxy)-3H spiro[isobenzofuran-1,9′-xanthen]-3-one). Yield 58%. IR (ATR, cm^−1^): 3525 (OH). 3068 (C-H)_Ar_. 2850, 2919 (C-H)_Aliph_. 1712 (C=O)_Ester_. 1503 (C=C)_Ar_. 1253 (O-C-O)_Ether_. ^1^H-NMR (400, CDCl_3_-d, δ ppm): 8.184 (d, *J* = 8 Hz, 1H, C-H_Ar_), 7.627 (m, *J* = 8 Hz 2H, C-H_Ar_), 7.215 (t, *J* = 8 Hz, 1H, C-H_Ar_), 6.836 (m, 3H, C-H_Ar_), 6.665 (d, *J* = 8 Hz, 1H, C-H_Ar_), 6.484 (d, *J* = 8 Hz,0 1H, C-H_Ar_), 6.403 (d, *J* = 8 Hz, 1H, C-H_Ar_), 3.995 (t, *J* = 8 Hz, 2H, C-H_Aliph_), 3.567 (m, 2H, C-H_Aliph_), 1.747 (m, 2H, C-H_Aliph_), 1.48 (m, 2H, C-H_Aliph_), 1.38 (m, 2H, C-H_Aliph_); 1.23 (m, 12 H, C-H_Aliph_), ^13^C-NMR (100 MHz, CDCl_3_-d, δ ppm): 184.93 (1C, C=O), 165.59 (1C, C_Ar_), 164.16 (1C, C-H_Ar_), 159.06 (1C, C-H_Ar_), 154.63 (1C, C-H_Ar_), 134.01 (1C, C-H_Ar_), 132.54 (1C, C-H_Ar_), 131.34 (1C, C-H_Ar_), 130.79 (2C, C-H_Ar_), 130.40 (2C, C-H_Ar_), 129.75 (1C, C-H_Ar_), 117.46 (1C, C-H_Ar_), 114.85 (1C, C-H_Ar_), 114.33 (1C, C-H_Ar_), 105.54 (2C, C-H_Ar_), 100.62 (2C, C-H_Ar_), 69.08 (1C, C-H_Aliph_), 65.81 (1C, C-H_Aliph_), 32.76 (1C, C-H_Aliph_), 32.76 (1C, C-H_Aliph_), 32.75 (1C, C-H_Aliph_), 32.55 (1C, C-H_Aliph_), 29.55 (1C, C-H_Aliph_), 29.47 (1C, C-H_Aliph_), 29.40 (1C, C-H_Aliph_), 29.37 (1C, C-H_Aliph_), 25.76 (1C, C-H_Aliph_). Elemental analysis; calculated: C, 74.08; H, 6.82; O, 19.10, found: C, 73.40; H, 6.14.

A solution of 0.01 mol of diglycolic anhydride in dry acetonitrile was added dropwise into a gently stirred solution containing 0.01 mol of 4-dimethylaminopyridine (DMAP), 0.01 mol of triethylamine and 0.01 mol of compound **7** in 20 mL of freshly distilled acetonitrile (Scheme 2). The mixture was stirred and refluxed under inert atmosphere for an additional three hours. The mixture was then cooled to room temperature before extraction with chloroform. The combined organic layer was washed with 15 mL of 5% of citric acid solution followed by 15 mL of saturated brine solution. The washing step was repeated for another two times and the organic layer dried with anhydrous magnesium sulfate. The chloroform solvent was removed by a rotary evaporator to produce orange oil product.

**1** 2-(2-((11-((3′-hydroxy-3-oxo-3H-spiro[isobenzofuran-1,9′-xanthen]-6′-yl)oxy)undecyl)oxy)-2-oxoethoxy)acetic acid. Yield 87%. IR (ATR, cm^−1^): 3525 (OH). 3074 (C-H)_Ar_. 2926, 2854 (C-H)_Aliph_. 1634 (C=O)_Acid_, 1732 (C=O)_ester_. 1453, 1421 (C=C)_Ar_. 1264 (O-C-O)_Ether_. ^1^H-NMR (400 MHz, CDCl_3_-d, δ ppm): 8.186 (d, *J* = 8 Hz, 1H, C-H_Ar_), 7.819 (m, 1H, C-H_Ar_), 7.692 (t, *J* = 8 Hz, 1H, C-H_Ar_), 6.882 (m, 3H, C-H_Ar_), 6.686 (d, *J* = 8 Hz, 1H, C-H_Ar_), 6.614 (d, *J* = 8 Hz1H, C-H_Ar_), 6.399 (d, *J* = 8 Hz, 1H, J = 8 Hz, C-H_Ar_), 6.23 (s, C-H_Ar_), 4.20 (s, 2H, C-H_Aliph_), 4.17 (s, 2H, C-H_Aliph_), 4.023 (t, *J* = 8 Hz, 2H, C-H_Aliph_), 3.857 (m, 2H, C-H_Aliph_), 1.725 (m, *J* = 8 Hz, 2H, C-H_Aliph_), 1.319 (m, *J* = 8 Hz, 2H, C-H_Aliph_), 1.339 (m, *J* = 8 Hz, 2H, C-H_Aliph_); 1.14 (m, 12H, C-H_Aliph_), ^13^C-NMR (100 MHz, CDCl_3_-d, δ ppm): 184.33 (1C, C=O), 171.54 (1C, C=O), 170.20 (1C, C=O), 165.82 (1C, C-H_Ar_), 163.90 (1C, C-H_Ar_), 158.82 (1C, C-H_Ar_), 154.13 (1C, C-H_Ar_), 150.47 (1C, C-H_Ar_), 136.12 (1C, C-H_Ar_), 131.05 (1C, C-H_Ar_), 130.57 (1C, C-H_Ar_), 130.48 (1C, C-H_Ar_), 129.82 (1C, C-H_Ar_), 129.11 (1C, C-H_Ar_), 117.14 (1C, C-H_Ar_), 114.69 (2C, C-H_Ar_), 111.26 (2C, C-H_Ar_), 105.01 (2C, C-H_Ar_), 101.29 (1C, C-H_Ar_), 83.23 (1C, C-H_Acid_), 79.59 (1C, C-H_Acid_), 68.43 (1C, C-H_Aliph_), 64.62 (1C, C-H_Aliph_), 29.54 (1C, C-H_Aliph_), 29.44 (1C, C-H_Aliph_), 29.40 (1C, C-H_Aliph_), 29.14 (1C, C-H_Aliph_), 28.91 (1C, C-H_Aliph_), 28.79 (1C, C-H_Aliph_), 28.54 (1C, C-H_Aliph_), 25.98 (1C, C-H_Aliph_), 25.83 (1C, C-H_Aliph_), Elemental analysis; calculated: C, 67.95; H, 6.19; O, 25.86, found: C, 67.54; H, 6.30.

### 4.3. Computational Details

All computations were performed with the G09 [52] system of programs using the B3LYP [53,54] hybrid functional and the split valence basis set with polarization and diffuse functions for heavy atoms. Vibrational frequency calculations were carried out to confirm that the optimized structures produce only positive frequencies, and the transition structures give one and only one large negative frequency. The Becke three-parameter [55,56] uses the nonlocal correlation provided by the LYP expression and the VWN [57] functional III for local correlation. The hybrid functional utilizes Becke 88 exchange functional [58]. All geometry optimizations were initially performed at the B3LYP/6-31+G(d,p) level with IEF-PCM [59] methanol continuum solvation model. Binding energies on small structures and complexes (fewer than ten heavy atoms) were obtained at B3LYP/6-311+G(2df,2p) level with the IEF-PCM methanol solvation model. The AOMix package [35] was used to analyze the nature of interaction between heteroatom and cation and in estimating the atomic valence and bond order (Mayer) [60] of the lactone C-O bond. GIAO [36] nuclear magnetic shielding calculations on carbon nuclei were performed with B3LYP/6-311G(2d,p) level to investigate the changes in shielding characteristics as the chromophore interacts with cation, lactone C-O bond breaks and conjugated π-network forms in the chromophore-cation complex. Natural Bonding Orbital (NBO) [34] calculations were performed, utilizing the built-in features in G09, to estimate orbital characteristics in chemical bonds of interest, i.e., the lactone C-O bond and the neighboring C-C bonds. PCA of computational and experimental data were performed using R programming language [61]. Score plots were obtained from the highest three principal components using commercial spreadsheet software.

## Figures and Tables

**Table 1:** Comparison of molecular properties in the interacting complexes of **1** and **1a** with Ca^2+^.

Parameter	^a^ 1-Ca^2+^	^b^ 1a-Ca^2+^
Ca Valence	0.43	0.41
Ca Charge	1.89	1.79
C_6_ *p*-Character	68.6	64.0
Ca-O_25_ TOP	0.092	0.090
C_7_-O_22_ BD	1.55	2.75
C_7_ Valence	4.90	3.56
O_22_ Valence	1.95	1.70

^a^ The optimized geometry in Figure 1c. ^b^ The optimized geometry in Figure 1b.

**Table 2:** Bond dissociation energies of complexes in methanol.

Dissociation Reaction	BDE (kcal mol^−1^)
^a^**2a**-Mg^2+^ **→ 2a** + Mg^2+^	18.2
^**a**^**2a**-Ca^2+^ **→ 2a** + Ca^2+^	9.1
^**a**^**2a**-Na^+^ **→ 2a** + Na^+^	4.3
^**a**^**2a**-K^+^ **→ 2a** + K^+^	3.5
^b^**4**-Mg^2+^ **→ 4** + Mg^2+^	29.5
^**b**^**4**-Ca^2+^ **→ 4** + Ca^2+^	11.8
^**b**^**4**-K^+^ **→ 4** + K^+^	7.2
^b^**4a**-Mg^2+^ **→ 4a** + Mg^2+^	53.7
^**b**^**4a**-Ca^2+^ **→ 4a** + Ca^2+^	27.2
^**b**^**4a**-K^+^ **→ 4a** + K^+^	12.6
^b^**2**-Ca^2+^ **→ 2** + Ca^2+^	6.5
^**b**^**4b**-Ca^2+^ **→ 4b** + Ca^2+^	11.6
^**b**^**2**–**4b**-Ca^2+^ **→ 2** + **4b**-Ca^2+^	5.6
^c^**3a**-Ca^2+^ **→ 3a** + Ca^2+^	22.1

^a^ with 6-311+g(2df,2p) basis set and ^b^ with 6-31+g(d,p) basis set. ^c^ **3a-**Ca^2+^ in acyclic form.

**Table 3:** Comparison of molecular properties in the complexes of phenol core **5** and its deprotonated form (**5a**) with **4**-Ca^2+^ and **4a**-Ca^2+^.

Parameter	^a^ 5-4-Ca^2+^	^b^ 5a-4a-Ca^2+^
Ca Valence	0.42	0.47
Ca Charge	1.88	1.86
C_4_ *p*-Character	69.5	69.1
C_7_-O_11_ TOP	0.412	0.028
C_7_-O_11_ Bond Order	1.34	1.25
C_7_ Valence	3.07	3.32
O_11_ Valence	1.85	1.81
C_4_ NMR	129.9	110.1
C_7_ NMR	96.5	100.8

^a^ The geometry in Figure 6d. ^b^ The geometry in Figure 6c.

**Table 4:** Photophysical characteristics of Fl-DGA (**1**).

[Fl-DGA] (mM)	3.2	1.0	0.3	0.1
λ_max_(nm)	544	544	544	544
I_m_/I_0_	1.0	5.2	2.1	1.2

*λ*_max_: maximum emission wavelength. *I*_m_/*I*_o_: ratio of maximum fluorescence intensity without metal cation.

## Data Availability

Not applicable.

## Supplementary Materials

The following supporting information can be downloaded at: https://www.mdpi.com/article/10.3390/molecules27196248/s1, Figure S1: Optimized geometries of diene-lactone **2** and its complexes; (**a**) diene-lactone **2**, (**b**) complex of **2** and Ca^2+^, (**c**) complex of **2** and K^+^, and (**d**) complex of **2** and Mg^2+^.; Figure S2: Parameter plots for diene-lactone **2** with B3LYP 6-311+g (2df,2p) and in methanol; (**a**) cation binding energy vs. HOMO stabilization, (**b**) C_3_-O_7_ bond distance vs. charge transfer, (**c**) C_3_-O_7_ bond distance vs. charge-to-radius ratio, and (**d**) C_3_ NMR vs. C_3_ p-character; Figure S3: Frontier orbitals of models; (**a**) HOMO-1 of ene-lactone **2a**, (b) LUMO+1 of **4a**-Ca^2+^ complex, (**c**) LUMO of **4a**-Ca^2+^ complex, (**d**) HOMO of diglycolic acid **4**, (**e**) HOMO-1 of **4**. (**g**) HOMO of diglycolate **4a**; Figure S4: Optimized geometries and parameter plots simple in methanol; (**a**) complex of enol **3** and Ca^2+^, (**b**) complex of enol **3** and K^+^, (**c**) **5a**-**4a**-K^+^ complex, (**d**) carbonyl CO bond distance vs. carbonyl O charge, (**e**) HOMO energy vs. carbonyl CO bond distance, and (**f**) C_3_-O_7_ bond distance vs. C_3_-O_7_ total overlap population; Figure S5: Structures of intermediates during formation of the acyclic form of **3a**-Ca^2+^.
