# Supplementary material for: Rational Design of a Fluorescent Chromophore as a Calcium Receptor via DFT and Multivariate Approaches
Source: Molecules. 2022 Sep 22;27(19):6248. doi: 10.3390/molecules27196248 (PMC9572636; doi:10.3390/molecules27196248)

Molecules

Supplement Information

Figure S1 . Optimized geometries of diene-lactone **2** and its complexes; (a) diene-lactone **2**, (b) complex of **2** and Ca^{2+}, (c) complex of **2** and K^{+}, and (d) complex of **2** and Mg^{2+}.

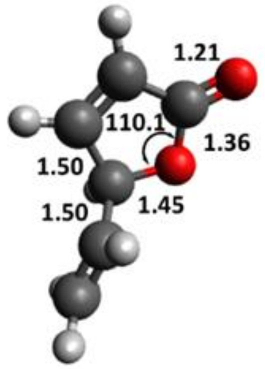

(a)

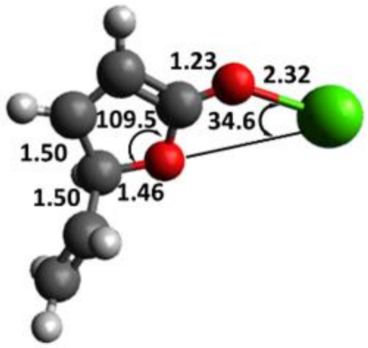

(b)

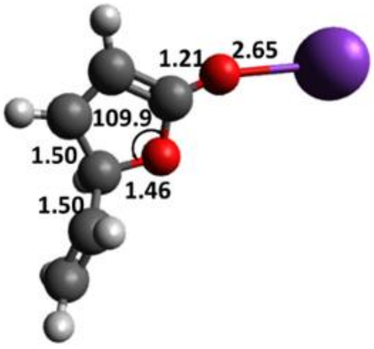

(c)

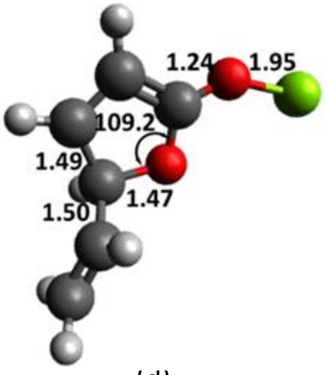

(d)

Figure S2. Parameter plots for diene-lactone **2** with B3LYP 6-311+g (2df,2p) and in methanol; (a) cation binding energy vs HOMO stabilization, (b) C_3-O_7 bond distance vs charge transfer, (c) C_3-O_7 bond distance vs charge-to-radius ratio, and (d) C_3 NMR vs C_3 *p*-character.

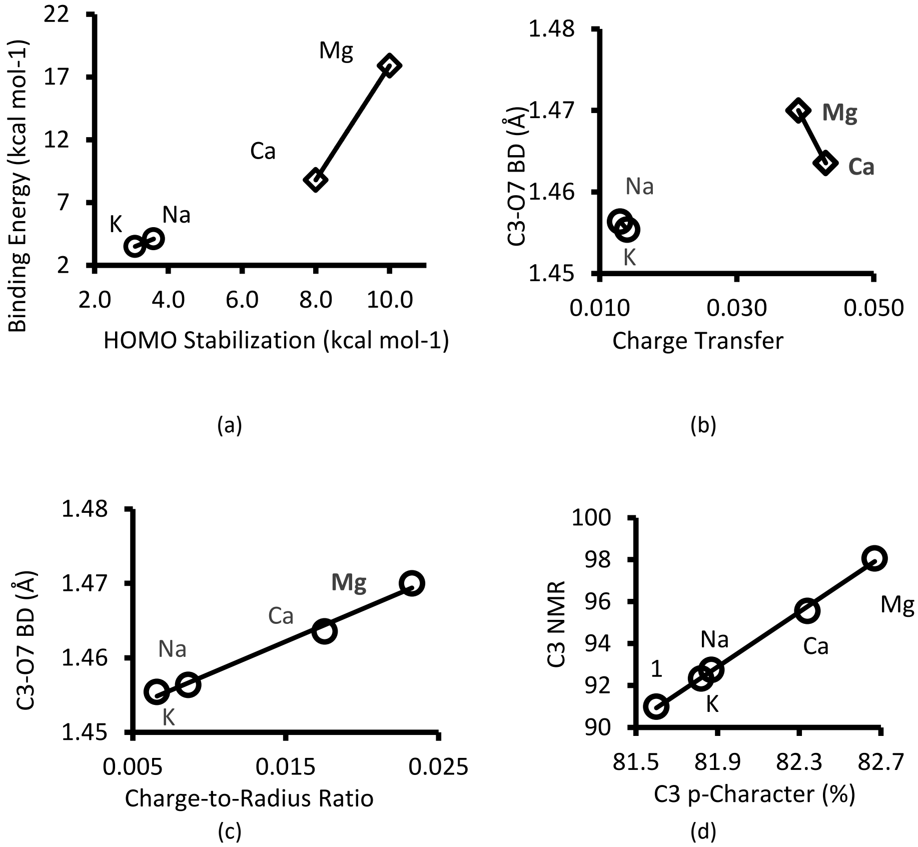

Figure S3. Frontier orbitals of models; (a) HOMO-1 of ene-lactone **2a**, (b) LUMO+1 of **4a**-Ca^{2+} complex, (c) LUMO of **4a**-Ca^{2+} complex, (d) HOMO of diglycolic acid **4**, (e) HOMO-1 of **4**. (g) HOMO of diglycolate **4a**.

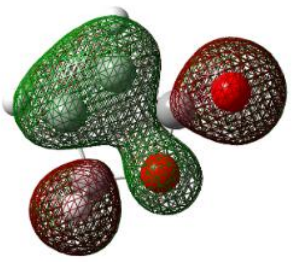

(a)

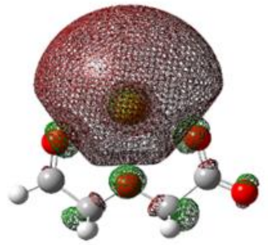

(b)

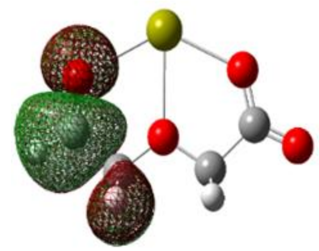

(c)

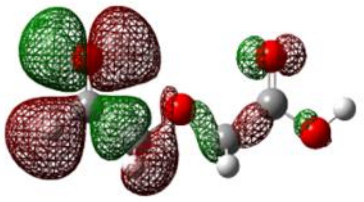

(d)

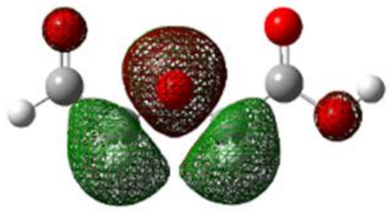

(e)

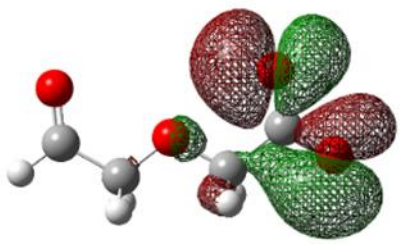

(f)

Figure S4. Optimized geometries and parameter plots simple in methanol; (a) complex of enol **3** and Ca^{2+}, (b) complex of enol **3** and K^{+}, (c) **5a**-**4a**-K^{+} complex, (d) carbonyl CO bond distance vs carbonyl O charge, (e) HOMO energy vs carbonyl CO bond distance, and (f) C_3-O_7 bond distance vs C_3-O_7 total overlap population.

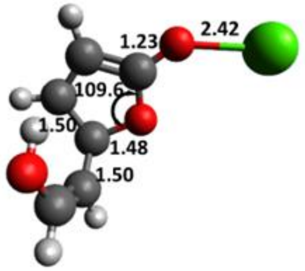

(a)

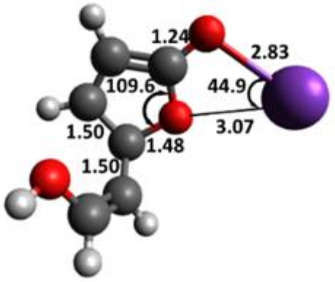

(b)

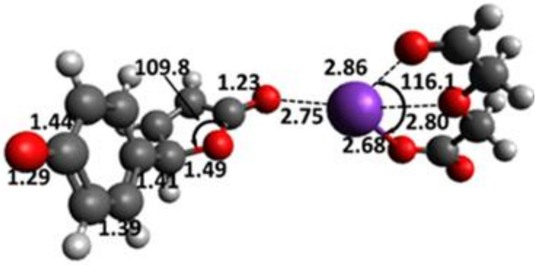

(c)

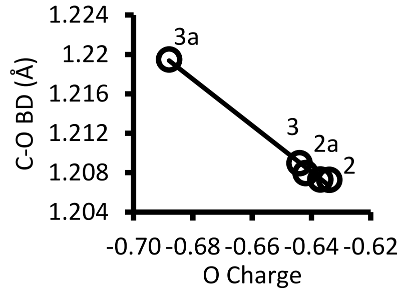

(d)

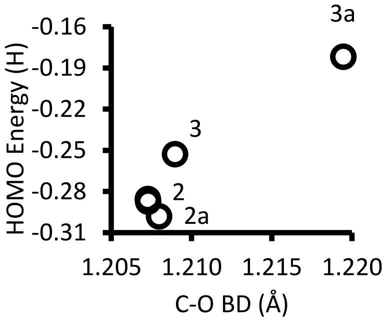

(e)

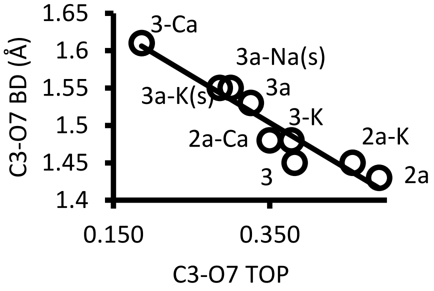

(f)

Figure S5. Structures of intermediates during formation of the acyclic form of **3a**-Ca^{2+}.

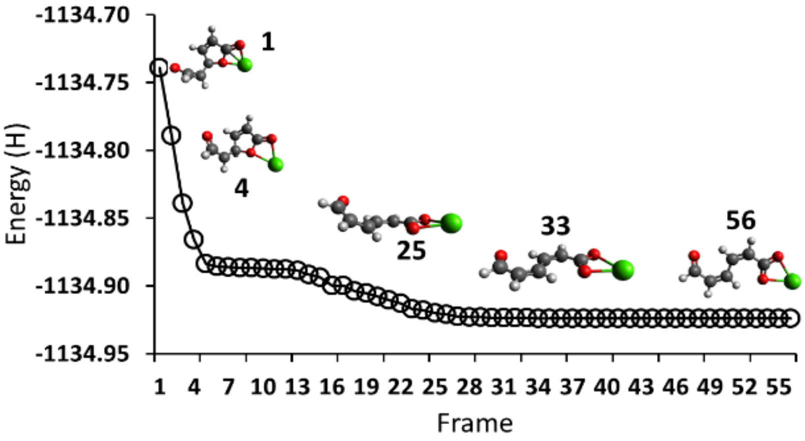

Supplement: Supplementary file 1 [file molecules-27-06248-s001.zip › molecules-1913184-supplementary.pdf]
